# Influence of geocoding quality on environmental exposure assessment of children living near high traffic roads

**DOI:** 10.1186/1471-2458-7-37

**Published:** 2007-03-16

**Authors:** Paul A Zandbergen

**Affiliations:** 1 Department of Geography, University of South Florida, 4202 E. Fowler Ave, NES107, Tampa, FL 33620, USA

## Abstract

**Background:**

The widespread availability of powerful geocoding tools in commercial GIS software and the interest in spatial analysis at the individual level have made address geocoding a widely employed technique in epidemiological studies. This study determined the effect of the positional error in street geocoding on the analysis of traffic-related air pollution on children.

**Methods:**

For a case-study of a large sample of school children in Orange County, Florida (n = 104,865) the positional error of street geocoding was determined through comparison with a parcel database. The effect of this error was evaluated by analyzing the proximity of street and parcel geocoded locations to road segments with high traffic volume and determining the accuracy of the classification using the results of street geocoding. Of the original sample of 163,886 addresses 36% were not used in the final analysis because they could not be reliably geocoded using either street or parcel geocoding. The estimates of positional error can therefore be considered conservative underestimates.

**Results:**

Street geocoding was found to have a median error of 41 meters, a 90^th ^percentile of 100 meters, a 95^th ^percentile of 137 meters and a 99^th ^percentile of 273 meters. These positional errors were found to be non-random in nature and introduced substantial bias and error in the estimates of potential exposure to traffic-related air pollution. Street geocoding was found to consistently over-estimate the number of potentially exposed children at small distances up to 250 meters. False positives and negatives were also found to be very common at these small distances.

**Conclusion:**

Results of the case-study presented here strongly suggest that typical street geocoding is insufficient for fine-scale analysis and more accurate alternatives need to be considered.

## Background

Advances in Geographic Information Systems (GIS), statistical methodology and availability of high-resolution georeferenced health and environmental data have created unprecedented opportunities for spatial epidemiology to investigate local geographic variation in disease [1]. GIS has become widely used to locate the study population by geocoding addresses, using proximity analysis of pollution sources as a surrogate for exposure, and integrating environmental monitoring data into the analysis of health outcomes [2]. As the capabilities of GIS have improved, address geocoding has become a very accessible research methodology and as a result the individual address is becoming a standard level of spatial investigation. Geocoding results are often used to determine the population or sub-population of which the study subjects are a part (for example, using census enumeration units) or to determine the relationship to other factors which vary spatially (such as air quality, distance to pollution sources, or proximity to health care services). Address geocoding can introduce bias and error [3] and the effect this has on the results of epidemiological studies has started to receive some attention in the literature [4,5]. This study adds to this research by exploring the effect of positional error in address geocoding using a case-study of the exposure potential of school children to traffic-related air pollution.

In the most common approach to geocoding addresses a street network is represented as street line segments that hold street names and the range of house numbers on each side of the street. Geocoding is accomplished by first matching the street name, then the segment that contains the house numbers and finally placing a point along the segment based on a linear interpolation within the range of house numbers. This technique is referred to as 'street geocoding'.

There are many potential problems with street geocoding, which have been well described in the literature [3-9]. Research on the quality of geocoding has emphasized a consideration of completeness, positional accuracy and repeatability [9]. Completeness is the percentage of records that can reliably be geocoded, also referred to as the match rate or hit rate. Positional accuracy indicates how close each geocoded point is to the 'true' location of the address. Repeatability indicates how sensitive the geocoding results are to variations in the street network input, the matching algorithms of the geocoding software, and the skills and interpretation of the analyst. These three factors combined describe the overall quality of the geocoding process. While each of these factors has been described in previous literature, the potential bias and error introduced by variability in match rates has received most attention [10,11]. The effect of positional accuracy of street geocoding on traffic pollution exposure estimates has received limited attention and is therefore the subject of this study.

Several studies have determined quantitative estimates of the positional accuracy of street geocoding by comparing the street goecoded locations with the 'true' location based on taking field measurements using a Global Positioning Systems (GPS) unit, using the centroid or boundary of the parcel, or determining the location of the residence using aerial photography. Estimates of 'typical' positional errors range from 38 to 75 meters [4,5,7,12-15] based on mean or median values. Results in urban areas are generally more accurate than in rural areas [4,14,15]. This suggests that the positional error of street geocoding can be substantial and needs to be characterized in a meaningful manner relevant to the use of the geocoded locations. In particular reference to epidemiological studies, when short distances are associated with health effects, the geocoding results must have a positional accuracy that is sufficient to resolve whether such effects are present [3].

Vehicular traffic related emissions are a major source of air pollution, especially in urban areas. Residential proximity to busy roads has been associated with health effects in children, in particular respiratory symptoms and asthma [16-29]. Several studies have also found associations between proximity to traffic and higher rates of childhood cancer [30-32], but not all studies have been conclusive in this regard [33,34].

Many studies have documented that the concentration of traffic pollution drops off rapidly with increasing distance from the road [26,28,35-44]. Concentrations are highest near roadways, decrease rapidly following an exponential function, and reach near background levels at approximately 300 to 500 meters from the road. Based on this strong spatial gradient in pollutant concentrations, measuring proximity of children's residences or school locations to major roads using GIS has become a widely employed alternative to actual exposure monitoring. In a typical analysis scenario, one or more buffer sizes are used to determine if geocoded locations fall within certain distances from major roads. Most studies use only a single buffer distance, including 100 meters [45], 150 meters [20,46], 169 meters [47], 229 meters [48], 300 meters [29] and 457 meters [33]; few studies have used multiple distances ranging from 30 to 300 meters [23,49,50]. While the use of discrete buffer distances has been criticized for not capturing the true distance-exposure relationship [51,52], their use is justified by the strong empirical evidence that pollutant concentrations follow a relatively predictable and rapid decrease with distance.

Studies on the effect of traffic-related air pollution have also considered traffic volume in the determination of environmental exposure conditions; adverse effects are observed for traffic counts starting at about 25,000 vehicles per day [19,47-53], which has become the lower exposure threshold used in studies that have modeled the potential exposure based on traffic counts and proximity [46,49,54].

The threshold values chosen for distance(s) and traffic volume are based on the result of epidemiological studies as well as studies monitoring the air dispersion of traffic-related pollutants. A final decision in the analysis is the selection of the specific spatial analysis metric to use. Distance to the nearest major roadway with a high traffic count per day is a commonly employed metric [20,23,46], although others have been utilized in epidemiological studies, such as the sum of traffic count within a buffer [48], distance-weighted traffic density [20,29,30,47,48], and traffic count at the nearest road [31]. Studies comparing these traffic metrics to actual exposure to traffic-related pollutants have been few [20], making the selection of proximity analysis technique somewhat arbitrary. The distance to major road metric has therefore been suggested as a reasonable, relatively easy-to-visualize metric for descriptive purposes [46]. Proximity to major roads is also computationally easy to estimate from data that is readily available, compared to the meteorological and traffic volume data required to model exposure conditions.

In summary, most studies on the exposure of children to air pollution from traffic have used relatively short distances of 50 to 500 meters to major roadways with daily traffic counts of 25,000 or more. Given these relatively short distances, the question arises whether the geocoded locations of children's residences are accurate enough to allow for this type of proximity analysis.

The accuracy of the results of the proximity analysis technique described above will depend in part on the positional accuracy of the input data [49,55]. At least four types of positional error can be identified:

1. *Positional error in street reference data*. This includes both the street network used for street geocoding as well as the street network used to determine vehicle counts (which are usually different). This positional error is closely related to the scale of the reference data. For example, data at a scale of 1:24,000 will be accurate to within 12 meters 90% of the time based on National Map Accuracy Standards (NMAS) [56]. The widely used Topologically Integrated Geographic Encoding and Referencing (TIGER) street data from the US Census Bureau meets the standard for 1:100,000 scale maps and will be accurate to within 50 meters 90% of the time, although the most recent versions of TIGER data are expected to be of greater accuracy [57,58].

2. *Positional error in representation of the road*. The road is normally represented as a single centerline, which may be a poor representation of the actual geometry of the road, which could consist of many lanes. This error will be small for minor roads but can be substantial for major highways.

3. *Positional error in the representation of the residences*. The residence is commonly considered as a point location, but this may not represent the actual building of interest. This error will be small for single family residences, but can be substantial for multi-family housing units.

4. *Positional error in geocoding*. This error is the distance between the street geocoded location and the 'true' location of the residence of interest.

These errors are potentially additive, presenting a major challenge to fine-scale analysis which relies on small positional error. Of the four types of errors above, only the first has received attention in the literature on the effects of traffic-related air pollution on children [49,55]. Both these studies determined the accuracy of using moderately accurate street reference data for geocoding by manually re-aligning it with higher quality reference data. Geocoding results were found to be very inacurate for analysis using short distances. The fourth type of error (positional error in geocoding) has been addressed by one recent study [5] in the context of traffic-related air pollution, although not specific to children. This study determined the potential misclassification of residences being located within 100 meters of a highway due to (modeled) positional errors in street geocoding. Misclassification in the form of false positives and negatives was found to be quite common, and increased for larger positional errors.

The objective of this study is to determine the influence of the positional error in street geocoding on the analysis of the effect of traffic-related air pollution on children. The other types of errors will be minimized by using very accurate street reference data. A very large sample of school children is used to allow for a proper characterization of the error distribution and its effect on analysis results.

## Methods

The study design relies on a comparison between the results of street geocoding and parcel geocoding. Parcel geocoding is used as a control and the distance between the street geocoded location and the parcel centroid is used as the estimate of positional error.

**Figure 1:**
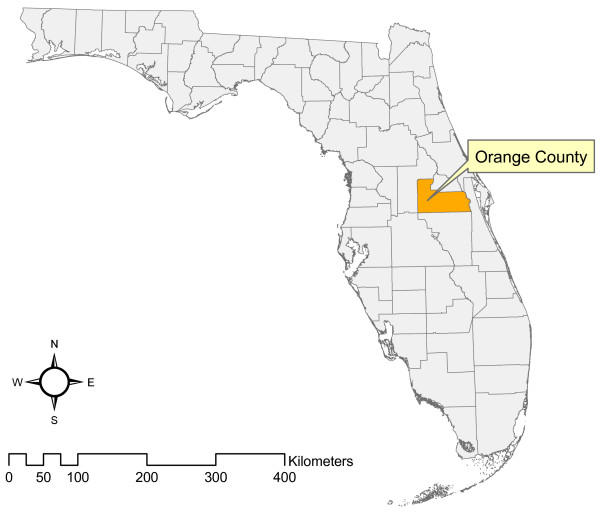
Study area location: Orange County, Florida.

Student enrollment records for 2005 were obtained from the Orange County School Board for all public schools in Orange County, Florida. The location of Orange County is shown in Figure 1. The largest City in Orange County is Orlando and the estimated total population of Orange County is estimated at 1,023,023 [59]. Student enrollment records contained the home residence of each student. These 163,886 addresses were street geocoded using a 1:5,000 street centerline network from Orange County for 2005 and parcel geocoded using a 1:2,000 parcel database of the Property Appraisers Office of Orange County for 2005, both using ArcGIS 9^®^. Geocoding parameters were set as follows: spelling sensitivity of 80, minimum match score of 80, no ties allowed. For both techniques manual interactive matching was used to identify reliable matches for records that did not produce a match score of 80 or higher in the automated geocoding process. A perpendicular offset of 8 meters was used in the placement of the street geocoded locations and is based on the typical width of the right-of-way of residential streets of 15 to 20 meters. The offset is used in an attempt to place the street geocoded location directly in front of the property instead of in the middle of the road. This distance is similar to values used in other studies that have employed street geocoding to determine the effects of traffic-related air pollution [5,46,50].

155,923 records or 95% of the total could be matched using street geocoding and 108,502 records or 66% could be matched using parcel geocoding. The much lower match rate for parcel geocoding is common since an exact match is required for the street number, while for street geocoding a match is obtained if the street number falls within the range of street numbers for a street segment without verification whether the exact street number exists or not. To characterize a potential bias introduced along urban/rural gradients, the study area was split up based on the population density of 5-digit ZIP codes. Areas with a population density of less than 250 people per square kilometer (based on 2003 estimates) were considered rural and the remaining areas were considered urban. Of the original 163,886 records 99% had a valid 5-digit ZIP code, so the comparison is limited to those 162,994 records. For those records with a valid 5-digit ZIP code 11% were considered rural and 89% were considered urban. Street geocoding match rates were 94% for rural areas and 96% for urban areas. Parcel geocoding match rates were 66% for rural areas and 67% for urban areas. These very similar values suggest that no bias was introduced due to differences in match rates along urban/rural gradients.

For the remainder of the analysis, only those records which could reliably be geocoded using both techniques were used (n = 104,865). The positional accuracy of the street level geocoded locations was determined by measuring the Euclidean distance between the street level geocoded point and the centroid of the associated parcel. The centroid was used instead of the property boundary since the centroid is expected to be a more accurate representation of the actual structure used as a residence. The distribution of the positional error was characterized using a cumulative frequency distribution and summary descriptive statistics.

Exposure potential to traffic-related air pollution was determined using proximity to roads with large traffic volume. A detailed road network for the State of Florida was obtained from the Florida Department of Transportation (FDOT) with Average Annual Daily Traffic (AADT) values for 2005 each road segment. The road network is compiled to meet the accuracy standards of a 1:24,000 basemap. Those road segments with an AADT of 25,000 or higher were selected for further analysis. For each geocoded residence the Euclidean distance to the nearest road segment was determined using ArcGIS 9^® ^for both the parcel centroids and the street geocoded locations.

Bias and error introduced by street geocoding was determined in several ways. First, the cumulative distribution functions of the proximity of children to roads with high traffic counts using street and parcel geocoding were compared to determine if street geocoding resulted in a consistently higher or lower number of children at-risk, in particular for short distances up to 1,000 meters. Second, the number of correctly and incorrectly classified children using street geocoding was determined using buffer radii of 50, 100, 150, 250, 500 and 1,000 meters. This required determining for each buffer radius which children actually reside within that distance (based on parcel centroids), which children were correctly classified as living within that distance using street geocoding (confirmed positives), which children were incorrectly classified as living further away (false negatives) and finally which children who actually reside outside that distance were incorrectly classified as living within that distance (false positives). 'Bias' is defined here as a consistent over- or under-estimation of the population of children at-risk. 'Error' is defined here as the occurrence of false negatives and false positives. The results of the two geocoding techniques were compared at each distance using measures of sensitivity and specificity, as well as odds-ratios.

**Figure 2:**
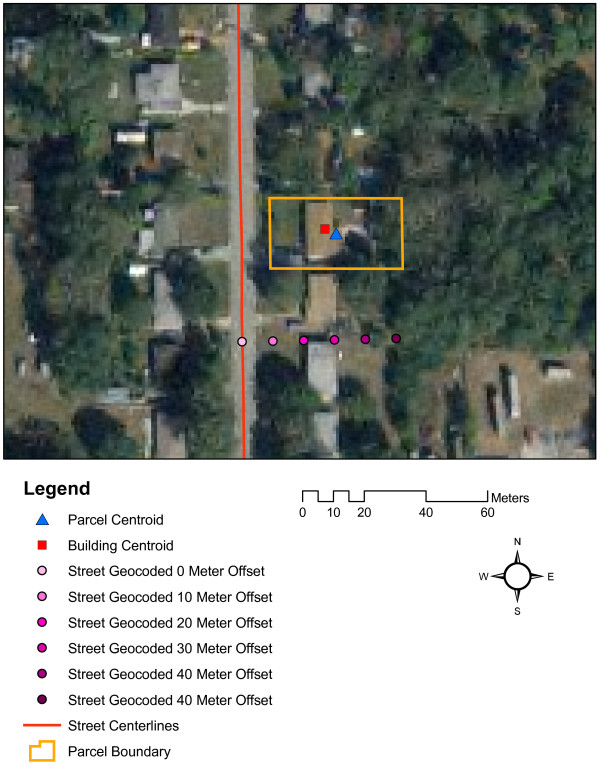
Illustration of the methodology to validate the positional accuracy estimates of street geocoding.

The analysis of the positional accuracy of geocoding in this study relies upon two assumptions: 1) that the parcel centroid is an accurate representation of the actual residential structure; and 2) that the chosen offset value used in the street geocoding is appropriate. These assumptions were tested using a random sample of 1,000 addresses. To test the first assumption, the location of the residential structure for these 1,000 addresses was determined (as a point location representing the structure's centroid) using 1-meter color digital orthophotos for 2004. The distance between parcel centroid and structure location was determined for each address as a measure of error of the parcel centroid technique. This distance was compared to the distance between the street geocoded location and structure location to determine if in fact the parcel centroid is a reliable technique to determine the error in street geocoding. To test the second assumption, the street geocoding was repeated using different offset values. A very small offset places the geocoded location very close to the street and therefore inevitably at some distance from the parcel centroid, while a larger offset potentially places the street geocoded location closer to the parcel centroid. Previous studies have reported a marginal effect of offset on the positional accuracy of geocoding [7,15], but this has not been addressed in relation to traffic related exposure estimates. The random sample of 1,000 addresses was street geocoded using offset values of 0, 10, 20, 30, 40 and 50 meters. Positional errors for each of these results were determined by measuring the distance of the street geocoded location to the structure location. Figure 2 shows an illustration of the analysis methodology used to test the assumptions.

## Results

**Figure 3:**
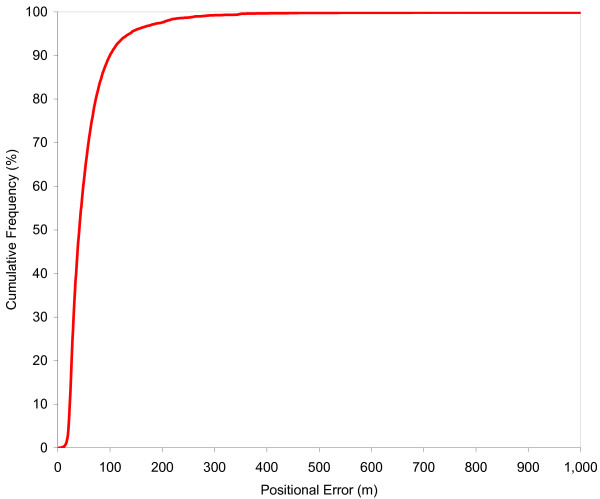
Cumulative distribution function of positional error in street geocoded locations of school children (n = 104,865).

Figure 3 shows the cumulative distribution function of the positional error in the street geocoding results and Table 1 provides descriptive statistics. The maximum positional error shown in Figure 1 is 1,000 meters while in reality a very small number of much larger error values occur. The positional error ranges from 1 to 32,356 meters, with a median of 41 meters. The 90^th^, 95^th ^and 99^th ^percentiles are 100, 137 and 273 meters, respectively.

**Table 1:** Summary statistics for the positional error (in meters) of street geocoded locations of school children in Orange County, Florida.

Sample	Min	Max	Mean	SD	Median	90^th ^%	95^th ^%	99^th ^%
Complete sample (n = 104,865) – distance to parcel centroid								
Offset 8 meters	1	32,356	66	435	41	100	137	273
Random sample (n = 1,000) – distance to structure location								
Parcel centroids	0	136	6	10	3	10	15	47
Offset 0 meter	17	3,367	61	115	43	102	138	292
Offset 10 meters	8	3,367	56	116	39	99	135	284
Offset 20 meters	0	3,367	52	117	37	99	133	284
Offset 30 meters	1	3,368	52	117	36	100	137	280
Offset 40 meters	2	3,368	56	116	38	103	138	275
Offset 50 meters	2	3,369	61	115	43	105	140	279

Table 1 also includes the results of the comparison between parcel centroids and the location of residential structures, as well as the results of the different offset values, both for a random sample of 1,000 addresses. The results confirm that parcel centroids provide a reliable measure of the location of the residential structure. Based on almost any of the accuracy statistics, the positional error of the parcel centroids is approximately one order of magnitude smaller than the error of the street geocoded locations. For example, the median error for the parcel centroids is 3 meters, while the lowest median error among any of the offset values considered is 36 meters. Similar comparison can be made for the other accuracy parameters. Relatively large positional errors in the parcel centroid locations are limited to a very small number of addresses, as indicated by the 99^th ^percentile of 47 meters. Results in Table 1 further confirm that the effect of offset value on the positional error is marginal. The accuracy statistics show very limited variation within the range of offset values considered. For example, the median error values ranges between 36 and 43 meters. Depending on which accuracy statistic is used, offset values of 10, 20 or 30 meters can be considered optimal, with slightly higher positional errors for offset values of 0, 40 and 50 meters. The consistency in the results for different offset values confirms that the analysis results are not sensitive to the chosen offset.

**Figure 4:**
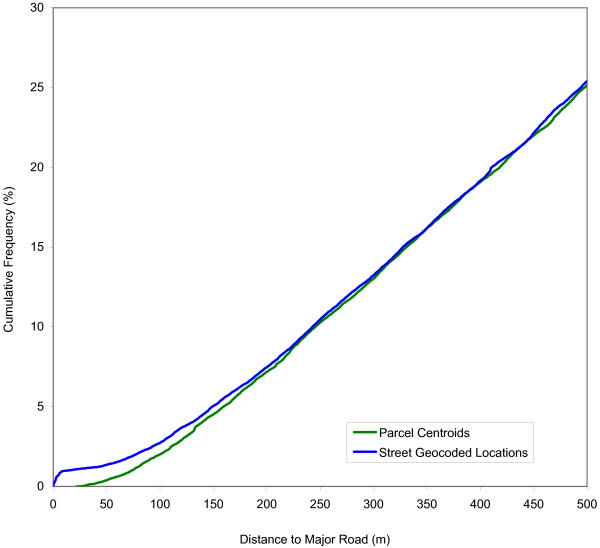
Cumulative distribution functions of the number of school children residing within a certain distance from a major road based on two geocoding techniques.

Figure 4 shows the cumulative distribution function of the number of school children residing within a certain distance from a high traffic density road for both the parcel centroids and street geocoded locations. Figure 4 only shows the smaller distances which are of most interest. The curve for street geocoded locations is consistently higher than for parcel centroids, indicating that street geocoded results in a consistent over-estimate of the number of at-risk children. This bias is most clearly observable at the shortest distances up to approximately 250 meters. While the observed bias is substantial, the total number of children living in close proximity to major roads is not extremely high: approximately 10% of all children live within 250 meters.

Table 2 shows the results of the analysis using the discrete buffer radii of 50, 100, 150, 250, 500 and 1,000 meters. This allows for a further characterization of bias and error. The results in Table 2 confirm a very strong bias towards an over-estimation of the number of children at risk when using street geocoding, particularly at short distances. For example, 391 children reside within 50 meters of a major road, while 1,413 are found using street geocoded locations, resulting in an over-estimate of 1,022. This means that the number of at-risk children is consistently estimated to be much higher than it actually is, and this presents a major bias in the analysis result.

**Table 2:** Bias and error in determining children at-risk based on proximity to major roads in Orange County, Florida.

Buffer Radius (m)	Number of children within buffer zone						Comparison measures		
	Parcels	Street	Parcels yes/Street yes	Parcels yes/Street no	Parcel no/Street yes	Parcel no/Street no	Sensitivity (%)^1^	Specificity (%)^2^	Odds Ratio (95% CI)^3^
50 meters	391	1413	151	240	1262	103212	39	99	0.28 (0.25 – 0.31)
100 meters	2090	2851	1392	698	1459	101316	67	99	0.73 (0.69 – 0.77)
150 meters	4717	5276	3899	818	1377	98771	83	99	0.89 (0.85 – 0.93)
250 meters	10729	10945	9704	1025	1241	92895	90	99	0.98 (0.95 – 1.01)
500 meters	26347	26597	25201	1146	1396	77122	96	98	0.99 (0.97 – 1.01)
1000 meters	54500	54614	53838	662	776	49589	99	98	01.00 (0.980 – 1.01)

^1^ Sensitivity is the percentage of parcel geocoded children residing within the buffer zone that were correctly classified using street geocoding.

^2^ Specificity is the percentage of parcel geocoded children residing outside the buffer zone that were correctly classified using street geocoding.

^3^ The odds-ratio is determined by comparing the parcel and street geocoded populations. The odds-ratio represents the odds that parcel geocoded addreses are within a given buffer radius, given that street-geocoded locations for the same address fall inside vs. outside the buffer radius. A value less than 1 indicates that the odds for the parcel geocoded population residing within the buffer radius is lower than for the street geocoded population

The observed bias is reflected in the changing odds-ratio, which is determined by comparing the at-risk populations using parcel centroids and street geocoding. A value less than 1 indicates that the odds for the parcel geocoded population residing within the buffer zone is lower than for the street geocoded population. For small buffer radii, the odds-ratio is much lower than 1, indicating a substantial bias. For larger distances the odds-ratio increases and starts to approximate a value of 1. At distances of 250 meters and larger, the 95% confidence interval for the odds-ratio includes 1, suggesting there is no longer any evidence of a statistical bias.

Table 2 also provides for a characterization of errors in the form of large numbers of false positives and negatives. For example, of the 391 children residing within 50 meters, only 151 were correctly classified as such using street geocoding (confirmed positives), and 240 were not (false negatives). The 1,262 children that were incorrectly identified as residing within 50 meters are false positives. This is further expressed in the measure for sensitivity, i.e. the percentage of parcel geocoded children residing within the buffer zone that were correctly classified using street geocoding. For the smallest buffer radius of 50 meters, the sensitivity is a low of 39%, suggesting street geocoding results are very inaccurate. Sensitivity gradually increases to 90% and higher for distance of 250 meters or more. Specificity, i.e. the percentage of parcel geocoded children residing outside the buffer zone that were correctly classified using street geocoding, is consistently high for all distance values considered because of the large number of confirmed negatives for all distances.

## Discussion and conclusion

The positional error in street geocoded locations was found to be very high relative to the accuracy requirements for this analysis: a median error of 41 meters and 90^th^, 95^th ^and 99^th ^percentiles of 100, 137 and 273 meters, respectively. These estimates are similar to those found in previous studies [4,5,7,9,12-15] and were obtained using a street network and parcel database of very high positional accuracy. Therefore, the observed errors are largely due to the geocoding process itself, not the underlying positional error in the reference data.

Comparisons with the actual locations of residential structures as determined from high resolution ortho-imagery confirmed the accuracy of using parcel centroids as a measure of the location of residences. The positional error in using parcel centroids is approximately one order of magnitude smaller than the positional error of street geocoding. The results for different offset values in the street geocoding resulted in marginal improvements in positional error, indicating the offset value is a very minor factor in determining positional accuracy of street geocoding.

The amount of bias and error introduced by the positional error in street geocoding is substantial. As a general rule, spatial data needs to be much more accurate than the minimum distance used in spatial analysis for the results to be meaningful [60,61]; this rule is clearly not met when utilizing the results of street geocoding in fine-scale analysis in the order of 100 meters. The large number of false negatives and positives, therefore, is to be expected given the magnitude of the positional error in street geocoding. The observed bias, however, was not expected. If the positional errors in street geocoding were completely random in their direction, the number of false negatives and positives for a given distance would be very similar and not result in the observed over-estimation of the number of children at-risk. The reason for the observed bias will be explored further.

**Figure 5:**
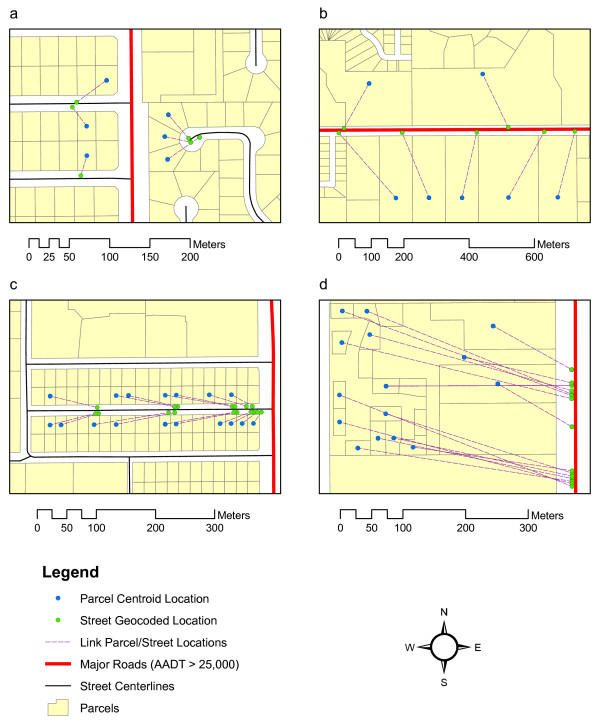
Examples of positional error in street geocoded locations of school children.

Figure 5 shows the geocoding results for selected areas which will be used to discuss several common scenarios. In each case, both the parcel centroid and the street geocoded locations are shown, connected with a line showing the association; the length of this line is the positional error in street geocoding for that address. Figure 5a shows a scenario where a number of parcels are in close proximity to a major road but the actual address is on a minor road. While for all addresses the positional error is fairly small, the street geocoded locations are further from the major road than the parcel centroids. This scenario represents the majority of false negatives encountered in the analysis. Figure 5b shows another scenario where the actual addresses are located on a major road; the street geocoded locations are placed 8 meters from the road based on the offset used, but the parcel centroids are located much further away. The example parcels in Figure 5b are relatively large in size, but the same effect also occurs with smaller parcels. This results in false positives but does not explain the observed bias since it simply represents the opposite of the scenario in Figure 5a. Closer inspection of the major roadways reveals that in many areas there are no residential addresses located along their segments: of the total sample of 104,865 addresses, only 918 (or less than 1%) are actually located on major roadways with traffic densities of 25,000 or more vehicles per day. The scenario presented in Figure 5a (parcels with their back or side facing a major road, but not their front) is therefore much more common than the scenario presented in Figure 5b. To explain the observed bias in the analysis result, Figure 5c illustrates a commonly observed scenario where all the street geocoded locations appear to be shifted to one side of the street. The reason for this can be seen by looking at the algorithm behind placing the locations; an address is matched by first matching the proper street segment based on name and address number range and then placing the address along this segment using linear interpolation within the recorded address range for the segment. In many cases the recorded address ranges assume rounding to intervals of 100 for a single segment, for example from 00 to 98 on the left and from 01 to 99 on the right. The actual range may be much smaller. In the example shown in Figure 5c, the actual range is from 2800 to 3018 on the left and from 2803 to3017 on the right while the recorded range in the street centerline database includes a range from 2800 to 3098 on the left and from 2801 to 3099 on the left. This results in a consistent shift of street geocoded locations to the start of the street segment with correspondingly large positional errors; this effect can be referred to as a "squeeze" towards one side of the street segment. This particular type of positional error has previously been reported as a common occurrence in street geocoding [3] but its effect has not been quantified in previous studies. In the context of the determination of proximity to major roads, this positional error has a particularly undesirable effect. Since many residential streets are collector streets for major roads and often have their starting address numbers at the major roads, the shift towards the start of the street segment results in consistent over-estimation of the number of children at-risk. This explains the observed bias in the analysis results, since the scenario observed in Figure 5c is very common throughout the study area.

The errors in address ranges are not unique to the street centerline data used in this study. For comparative purposes, a TIGER street network for 2000 and a StreetMap USA street network for 2005 were obtained; for 25 randomly selected segments where the address range in the street centerline data was known to be incorrect (i.e. assumed to be rounded to 100 while the range in the parcel database was smaller), both the TIGER and StreetMap USA data revealed identical errors.

Finally, Figure 5d shows a scenario where parcels are located at a substantial distance from the road on which their address is located; this occurs in areas such as mobile home parks. The positional error of the street geocoding is very large (> 100 meters) and introduces further bias, but this scenario is much less common than the scenario presented in Figure 5c.

Positional errors in street geocoding were found to introduce substantial bias and error in the analysis of the effects of traffic-related air pollution on children. The magnitude of the bias is substantial at small distances, but is no longer observed at distances of 250 meters or more. A substantial number of false positives and negatives is observed at small distances, but sensitivity increases to 90% or higher at distances of 250 meters or more. This strongly suggests that typical street geocoding is insufficient for fine-scale analysis at a level of several hundred meters or less, since this produces very inaccurate results. This study confirms the results from previous studies [4,5] that the positional error in street geocoding introduces substantial misclassification in environmental exposure assessments at short distances. The strong bias observed in this study for traffic-related air pollution, however, has not been previously documented.

Alternatives to street geocoding need to be considered, including parcel-based geocoding, address point geocoding, the use of ortho-imagery and field observations using GPS. So far, only one recent study investigating the effects of traffic-related air pollution on children has employed this type of accurate geocoding [62], but this is expected to become a more common practice.

The findings from this study are based on a single geographic area (Orange County, Florida) and are therefore not automatically generalizable to other areas, despite the large sample size. The first limitation is that the street geocoding technique employed in this study is typical for the United States. Other jurisdictions may employ different geocoding techniques which cannot be assumed to result in similar positional errors. The second limitation is that only a single street geocoding technique was used. The reference data used, however, is of high positional accuracy (1:2,000 street centerlines), and using reference data of lower positional accuracy (such as 1:100,000 TIGER streets) would likely results in larger positional errors. The third limitation is that a substantial portion of the original addresses could not be geocoded using both parcel centroids and street centerlines. Low match rates are common for parcel geocoding, in particular for multi-family residences. This has most likely skewed the results towards the highest quality data, i.e. addresses that could be reliably geocoded and single-family residential housing. This suggests the real possibility that the error estimates obtained from the final sample of locations are very conservative. The fourth limitation is that the positional accuracy of geocoding varies across urban/rural gradients. While the study area includes both urban and rural areas, it is predominantly urban and differences across these gradients were not explicitly examined. For areas that are mostly rural, the positional error of street geocoded will typically be larger than for urban areas, but the effect this has on the exposure to traffic-related air pollution has not been investigated. The fifth limitation is that of the original sample of 163,886 addresses 36% were not used in the final analysis because they could not be reliably geocoded using either street or parcel geocoding. The estimates of positional error can therefore be considered conservative underestimates.

A final limitation of this study is that similar data (street centerlines, parcel boundaries and/or centroids) with geocoding capabilities are not available for all areas in GIS-compatible format. While this type of data is becoming more common across local jurisdictions in the United States, the methodology employed here may not be replicable in all areas.

The widespread availability of powerful geocoding tools in commercial GIS software and the interest in spatial analysis at the individual level have made address geocoding a widely employed technique in epidemiological studies. While some of the limitations of street geocoding have been addressed in recent review articles in public health and epidemiology journals [3,63], most studies have employed street geocoding without much consideration to its inherent limitations. Match rates have received most recognition, and the positional error has been assumed to be small in magnitude and random in its effect on analysis results. This study has shown that the positional error in street geocoding is neither small nor random, and that caution in the use of street geocoding results for epidemiological studies is warranted. Street geocoding is very appealing as a data processing step since it provides a high degree of automation, but the results are not accompanied by accuracy estimates for its quality other than match scores. The use of street reference data of high positional accuracy and currency is no guarantee the positional accuracy of street geocoding will be sufficient for fine-scale spatial analysis.

## Competing interests

The author(s) declare that they have no competing interests.

## Authors' contributions

PAZ obtained and processed all the data, performed the street geocoding, as well as the distance analysis and the statistical comparisons, and wrote and revised the text.

## Pre-publication history

The pre-publication history for this paper can be accessed here:


